# Competence-Based Curricula in the Context of Bologna and EU Higher Education Policy

**DOI:** 10.3390/pharmacy5020017

**Published:** 2017-03-26

**Authors:** Howard Davies

**Affiliations:** European University Association, Avenue de l'Yser 24 Ijserlaan, Brussels B-1040, Belgium; howard.davies@eua.be; Tel.: +44-7780-700-648

**Keywords:** competence, profession, curriculum, policy

## Abstract

At the turn of the century European higher education policy became twin-track. The Bologna Process was launched and ran alongside developments in European legislation. Both tracks displayed a preoccupation with competences, in relation both to citizenship and to labour market needs. Scrutiny of important policy texts (Key Competences, the European Qualifications Framework, ECTS, the Bologna three-cycle degree structure) shows that ‘competence’ has never been given a precise and secure definition. Only very recently has the term entered the discourse of EU legislation on the recognition of professional qualifications. Current work on competence-based curricula in sectoral professions, including pharmacy, has helped bring the two policy tracks into closer alignment. The examples of competences identified in specific professional contexts can assist EU and Bologna policy-makers as they confront future challenges.

## 1. Introduction

European higher education policy follows two tracks. The first, in historical terms, is the vision elaborated over many years by the European Commission. A host of Communications and working documents have been produced, which may or may not have been subsequently enshrined in legislation. This would have depended on their acceptability to the legislative bodies—Council and Parliament—but also on the degree of legal competence enjoyed by the European Community or the European Union at any given moment in time. 

Since the Treaty of Maastricht in 1992, education has become much more strictly the province of Member States (MSs); the capacity of action of the EU institutions is now that of a ‘complementary competence’, undertaking at EU level that which cannot be achieved by MSs acting independently.

The second track is the Bologna Process, an inter-governmental action programme dating from the Sorbonne Declaration of 1998 and the Bologna Declaration of 1999. By 2010, the Process had put in place the European Higher Education Area (EHEA). The EHEA is effectively co-regulated by the 48 signatory countries (or regions of countries) together with European-level bodies representing social partners and sectoral stakeholders (institutions, students, quality assurance agencies). It has a legal base, the Lisbon Recognition Convention (LRC), although in practice the relevant legislation is enacted by the signatory countries within their own territories. 

The two tracks are not independent of each other. The European Commission participates in summit meetings and is also a member of the Bologna Follow-Up Group (BFUG) which manages Bologna Process business between the bi- or triennial meetings of the 48 ministers. It has funded a number of the Bologna Process initiatives. 

But neither are the two tracks fully integrated or geo-politically congruent. The Bologna Process covers countries from Iceland to Russia, from Norway to Armenia. EU legislation extends to the 28 MSs and the three countries in the European Economic Area—Iceland, Liechtenstein and Norway. Their relationship, however, has evolved through time, to the extent that it is now possible to speak of a gradual convergence, at least as far as the recognition of professional qualifications and the development of competence-based curricula are concerned.

For over the past ten years, a number of professions have embarked on competence-based curricula as a way of consolidating both professional expertise and public trust. In many instances—medical doctors, dentists, nurses, pharmacists and architects—the emphasis has been on refocusing the basic training prescribed by EU legislation on the recognition of professional qualifications [1]. In doing so, the professions—and notably the academics in the professions—have worked within the parameters of the Bologna Process. Their work has been located at the point of convergence of two related trends: the shift from teacher-centred training to student-centred learning, orchestrated by the Bologna Process; and the determination to integrate the EU labour market following the financial crisis of 2008. 

## 2. Europe Pre-Bologna

EU employment policy did not emerge fully-formed in the aftermath of 2008. Article 57.1 of the Treaty of Rome (1957) laid the foundation stone for current legislation on the recognition of professional qualifications, based on the free movement of citizens and the drive to create a Single Market. While it led to the raft of Directives on seven sectoral professions (including that of the pharmacist) in the late 1970s, it did not represent the birth of a higher education policy. That particular initiative had already been taken, relatively independently, in the early part of the same decade. Anne Corbett offers a detailed and insightful account of what she refers to as the ‘creation of a policy domain’ [2] by the ‘policy entrepreneurs’ who created the ERASMUS programme of student and staff mobility and the COMETT support for university-enterprise partnerships. 

In general, however, European higher educational policy statements [3] in the late 1980s and early 1990s mainly concern mobility, quality assessment, and distance learning. Labour market considerations are reserved for the vocational education and training (VET) sector. The Treaty of Maastricht in 1992 checked the flow of educational initiatives by confining the European institutions to a complementary role, the principal responsibility for higher education resting with MSs. This made it more challenging to implement the conclusions of the Study Group on Education and Training set up by Commissioner Cresson in 1995, which stressed the importance of adopting a ‘strategy of continually raising competence levels’ [4]. 

It was the perceived need to re-assert a transnational policy frame that prompted France, supported by Germany, Italy and the UK, to resort to inter-governmental action in 1998. This was the Sorbonne Declaration, which one year later mutated into the Bologna Process. It proposed concerted structural change, the facilitation of mobility by credit accumulation and transfer, and a framework of lifelong learning. It was then that the flow of policy-making divided into the two tracks described above. Between them, the ability to cross-refer was not strong. The Bologna Process involved ministers of education in a series of commitments, which those from European Community MSs were able to echo in meetings of the Education Council. However, coming from governments organised in different ways and according to different priorities, they enjoyed varying degrees of collaboration, at European level, with their peers who sat in the Competitiveness Council and who were responsible for research, national economies and labour markets. It was left to the Commission to take up the challenge put down by Edith Cresson’s Study Group and to address the question of competences.

## 3. Key Competences in the European Union

The ambitious Lisbon Strategy, with its oft-quoted aim—“to become the most dynamic and competitive knowledge-based economy in the world by 2010 capable of sustainable economic growth with more and better jobs and greater social cohesion and respect for the environment”—languished at first. The Wim Kok report of 2004 [5] subsequently left no room for doubting that the strategy had run into difficulty. The European Commission responded with a Communication on *Mobilising the brainpower of Europe: enabling universities to make their full contribution to the Lisbon strategy* [6] in 2005. 

Yet already in 2003, in their joint interim report on *Education and Training 2010: the success of the Lisbon Strategy hinges on urgent reforms* [7], the Commission and the Council had bemoaned the fact that ‘nearly 20% of young people fail to acquire key **competences** [8]’:

Everyone needs to acquire a minimum set of **competences** in order to learn, work and achieve fulfilment in a knowledge-driven society and economy. They include traditional key **competences** (reading, writing and numbers) and the newer ones (comprising foreign languages, entrepreneurship, interpersonal and civic **competences**, and **competences** in the new information and communication technologies). However, in the fundamental domain of reading, 17.2% of young Europeans aged under 15 do not have the minimum **competence** required […]

This was to become a familiar refrain. In 2006, the Commission duly published a list of *Key Competences* which was then adopted as a formal Recommendation [9]. Ten in number, the competences were the following:

- communication in the mother tongue;
- communication in foreign languages;
- competences in maths, science and technology;
- digital competence;
- learning to learn;
- interpersonal, intercultural and social competences, and civic competence;
- entrepreneurship;
- cultural expression.

They were to be attained by all citizens by the end of their initial education and training. Without them, the scope for personal fulfilment and social cohesion would be curtailed. It was nevertheless labour market considerations that drove the Commission’s initiative.

The Lisbon Agenda of 2000 had been predicated on the principle that competitiveness and cohesion were not mutually exclusive, as well as on the presumption that the EU would thrive by generating knowledge which could be monetised by its transformation into manufactured goods in low-wage economies, notably China. If Europe were to sustain itself as a high-skill, high-wage society, its citizens would have to be educated and trained to the appropriate level—on a lifelong basis. Hence the generic character given to the competences: they were multifunctional and transferable. 

The competences were not, however, entirely free-floating. If left to the political will of MSs acting in their own pressurised policy environments, the prospect of uniform application would be at risk. It was necessary to tie the competences into the overarching framework of the European Qualifications Framework for lifelong learning [10] (EQF). 

This was done primarily by ensuring that both Recommendations—Key Competences and EQF—were based on compatible definitions of competence. This was a significant step. For native speakers of English, the notion of competence features in common parlance; speakers are nevertheless aware that it implies value judgements which can vary widely. 

A workable definition in Euro-English would have to be sufficiently supple to accommodate the intuitions of *compétence*, *Kompetenz*, and so on. The solution adopted was to give it the status of an umbrella concept, under which a bundle of attributes could be gathered:

- In the context of the Key Competences, a **competence** is a ‘combination of knowledge, skills and attitudes appropriate to the context’ [9] (Annex to Recommendation 2006/962/EC).
- In the EQF, ‘**competence** means the proven ability to use knowledge, skills and personal, social and/or methodological abilities, in work or study situations and in professional and personal development. In the context of the European Qualifications Framework, **competence** is described in terms of responsibility and autonomy’ [10] (Annex to Recommendation on EQF).

## 4. Competences in EU Legislation on the Recognition of Professional Qualifications

How do these developments translate into the Directives which apply to the sectoral professions? Only indirectly, it must be said, if at all. With respect to pharmacy [11], the term ‘competence’ appears nowhere. 

Article 45.2 of Directive 2005/36/EC lists seven ‘activities’:

(a) preparation of the pharmaceutical form of medicinal products;
(b) manufacture and testing of medicinal products;
(c) testing of medicinal products in a laboratory for the testing of medicinal products;
(d) storage, preservation and distribution of medicinal products at the wholesale stage;
(e) preparation, testing, storage and supply of medicinal products in pharmacies open to the public;
(f) preparation, testing, storage and dispensing of medicinal products in hospitals;
(g) provision of information and advice on medicinal products.

The amended Directive 2013/55/EU subsequently re-phrased (e) to read: ‘supply, preparation, testing, storage, distribution and dispensing of safe and efficacious medicinal products of the required quality in pharmacies open to the public’. It amplified (f) to read: ‘preparation, testing, storage and dispensing of safe and efficacious medicinal products of the required quality in hospitals’. It also amplified (g) to read: ‘provision of information and advice on medicinal products as such, including on their appropriate use’. Finally, it added three further ‘activities’:

(h) reporting of adverse reactions of pharmaceutical products to the competent authorities;
(i) personalised support for patients who administer their medication;
(j) contribution to local or national public health campaigns.

‘Activity’, however, is a term which appears in neither of the two definitions of competence cited earlier. Moreover, it is clear that only effective safeguards will prevent incompetent persons from engaging in the activities listed. To this end, the Directive prescribes appropriate supplementary professional experience. Article 44.3 renders the practice of the activities conditional upon ‘the following knowledge and skills’:

(a) adequate knowledge of medicines and the substances used in the manufacture of medicines;
(b) adequate knowledge of pharmaceutical technology and the physical, chemical, biological and microbiological testing of medicinal products;
(c) adequate knowledge of the metabolism and the effects of medicinal products and of the action of toxic substances, and of the use of medicinal products;
(d) adequate knowledge to evaluate scientific data concerning medicines in order to be able to supply appropriate information on the basis of this knowledge;
(e) adequate knowledge of the legal and other requirements associated with the pursuit of pharmacy.

With the exception of the few amendments made in 2013, the specified ‘activities’, as well as the ‘knowledge and skills’, follow the wording of Article 1 and 2 of Directive 85/432/EEC [12], wording which in 2005 was twenty years old. Only minimal scrutiny is required to ascertain that the five bodies of knowledge make reference to one single elliptically expressed ability and to no ‘skills’ whatever. 

The wording now goes back thirty-two years—but the preoccupation with competence-based curricula is comparatively recent. How is it that academic and professional bodies in sectoral professions have only latterly begun to concern themselves with competence, when they have long been exercised over the relationship of theory to practice?

Partly the answer lies in their anxiety about patient safety, which has grown as a result of demographic trends and the expansion of patient and professional mobility across EU internal borders. Partly it is due to the influence of the Bologna Process. 

Thanks to its mobility instruments—notably the Diploma Supplement—Bologna brought a transparency to curricula which had not existed before. Professional mobility, although underwritten by Directives, had previously operated on the basis of trust. While this trust was not wholly blind, it appeared not to prompt scrutiny of the content of ‘foreign’ curricula. But as the EU enlarged from 2004 onwards, and as the volume of cross-border professional and patient mobility increased, healthcare stakeholders grew more sensitive to the need for the effective quality assurance of training programmes—their curriculum development, their delivery, and their accreditation. 

Problems then became apparent and, with them, a certain resistance to recognition. A report commissioned by the European Parliament in 2009 repeatedly stressed MSs’ lack of trust in each other’s education systems. It pithily concluded that ‘if the MSs could trust each other’s education systems and believe that a child nurse is well educated in the EU, regardless of the formal degree he or she has obtained, there might be fewer problems with recognition of professional qualifications’ [13] (p. 65).

The lack of trust helped prompt the Commission make provision in the amended Directive for ‘common training frameworks’ (CTF). Article 49a.1 allows training programmes designed by one third of Member States (i.e., currently ten) to be subject to automatic recognition, if based on a ‘common set of minimum knowledge, skills and **competences** necessary for the pursuit of a specific profession’ or of a specialty related to one of the sectoral professions. Hospital pharmacists fall into the latter category. Led by the European Association of Hospital Pharmacists (EAHP), they currently lead the field in the development of a CTF and are mapping the competences required in the advanced practice of hospital pharmacy in Europe [14].

Two factors are of note here. First, the amended Directive requires all CTFs to be based on the levels of the EQF, in order to ensure readability across the countries that have referenced their national qualifications frameworks to it. 

Secondly, the concept of competence now sits alongside knowledge and skills as an apparently separate category. Notwithstanding the scrubbing to which all EU legal texts are subject, ‘competence’ has lost its umbrella function as well as, accordingly, its utility as a term with an established legal usage. 

Loss of textual cohesion is not quite the same as incoherence, since some sense clearly survives. The looseness of expression is nonetheless worrying, particularly in view of recent developments which show the European Commission to be adopting an ever more ‘technicist’ approach to education and training in its promotion of employability. 

## 5. The Potential for Competence-Based Curricula in Pharmacy

Despite the introduction of CTFs, it remains true that the ‘core’ pharmacy Articles of the Directive do not explicitly encourage the development of competence-based curricula. But to what extent do they inhibit it? Recital 25 alludes to the ‘coordinated minimum range of activities’ to be covered in pharmacy training, clearly indicating that MSs may go beyond the minimum. They may, therefore, choose to require additional activities, which they may frame in terms of competences if they so choose. 

Furthermore, the Directive, while prescribing a minimum of five years full-time training, does not lay down a precise number of hours. Nor does it, in Annex 5.6.2, give a precise quantification of the required ‘balance’ between theoretical and practical work. Moreover, it allows the five years to be expressed in ECTS credits, which embrace contact hours, projects, practical work, placements and private study. 

On the face of it, it would appear—at least to the non-pharmacist author of this article—that EU legislation puts no impediment in the way of competence-based curriculum designers. They may proceed with as much freedom as their national regulatory framework allows. To secure a firmer transnational structure for collaboration, they have three recourses: first, the CTFs already mentioned: secondly, the review of the Directive in 2019; thirdly, applying pressure to the Bologna Process to build stronger consensus around the concept of competence.

## 6. Competences in the Bologna Process

To what extent is the unstable definition of competence mirrored in its occurrences in the Bologna Declaration of 1999 and in the eight ministerial communiqués which have followed it [15]? They reveal an initial focus on competences associated with citizenship. This focus never entirely disappears, but weakens as the emphasis on lifelong labour market relevance grows. At the most recent ministerial conference—in Yerevan in 2015—competence featured in its widest range of reference: citizenship, lifelong employability, international mobility. 

The Berlin formulation of 2003 spoke of a qualifications framework based on workload, level, learning outcomes, competences and profile, regarding learning outcomes and competences as different categories. It was historically significant, because it was in response to this ministerial communiqué that an informal joint Quality Initiative group developed the Dublin Descriptors in 2004. 

The Descriptors set out the expected attributes of students who have successfully completed courses at short cycle, Bachelor, Master and doctoral levels. The term ‘competence’ is not used systematically; surprisingly, it appears only at Bachelor level, where it means that which may be appraised through the medium of the assessment process. Competence here has a retrospective reference—while the learning outcome has the prospective character of a result yet to be obtained.

The Bologna ministerial communiqués have a flavour all their own. They have to establish continuity between the summit meetings. They have also to express a unanimity which, given the rising number of signatory countries, tends to be couched in generalities rather than specifics. The growing emphasis on employability from 2005 onwards, in parallel with the policy statements of the EU, is clearly discernible, and yet a consecutive reading of all the communiqués yields an overwhelming sense of repetitiousness. Despite this, they provide no stable definition of competence.

Greater hopes might be placed in the Bologna Follow-Up Group (BFUG), which manages the Process on a self-regulatory basis in the intervals between the ministerial summits. BFUG includes stakeholder bodies capable of giving the debates a stronger bottom-up character and a greater potential to tap into the thinking of the higher education institutions, their staff and their students. Yet it, too, falls short of providing clear definition: ‘learning outcomes are understood in their broadest sense and, in the case of the Dublin Descriptors and the Tuning project, include **competences**. Within some discourses, **competences** may have a more precise meaning, for example, in some assessment contexts they are associated with the performance of work-related tasks’ [16] (p. 41).

In the Tuning project, meanwhile, competence reverts to the umbrella function that it enjoyed in the EU’s Recommendation on Key Competences. Learning outcomes are subsumed within it, rather than standing apart as a separate category.

**Competences** represent a combination of attributes (with respect to knowledge and its application, attitudes, skills and responsibilities) that describe the level or degree to which a person is capable of performing them. […] In this context, a **competence** or set of **competences** means that a person puts into play a certain capacity or skill and performs a task, where he/she is able to demonstrate that he/she can do so in a way that allows evaluation of the level of achievement. **Competences** can be carried out and assessed.

[17] (p. 69)

## 7. Competences in the European Credit Transfer and Accumulation System (ECTS)

ECTS was funded and developed on a pilot basis by the European Commission in the early years of the ERASMUS programme. Three versions of its Users’ Guide have been published, the most recent of which (2015) was drafted by an ad hoc working group [18], chaired by the Commission but located within the framework of BFUG. What do the Users’ Guides tell us of the nature of competence? [19].

The first edition in 2005 stuck close to the early Tuning position, presenting competences as bundles of attributes, while setting learning outcomes at a higher order of complexity—being sets of competences, or effectively bundles of bundles. The third edition (2015) reiterated the 2009 Glossary entry, reproducing the EQF definition, but adding a statement to the effect that ‘learning outcomes express the level of competence attained by the student and verified by assessment’ [20] (p. 22). 

This assertion drew an immediate rebuttal from two of the most respected authorities on learning outcomes, Declan Kennedy and Marion McCarthy (both of University College Cork). They point out that there is a widely accepted definition of a learning outcome: a statement of what a learner is expected to know, understand and/or be able to demonstrate after completion of a process of learning, but ‘there is no agreement in the literature on the meaning of competence’ [21] (p. 3). Indeed, they say, there is significant confusion, for which they hold the Tuning project responsible.

Kennedy and McCarthy recommend the EQF definition, re-stated below, because it is that which should apply to Common Training Frameworks developed in the framework of the amended Directive. It is therefore the definition which should be borne in mind by designers of competence-based curricula for sectoral professions.

**Competence** means the proven ability to use knowledge, skills and personal, social and/or methodological abilities, in work or study situations and in professional and personal development. In the context of the European Qualifications Framework, **competence** is described in terms of responsibility and autonomy.

It is evident that it is easier to identify competences required in context than to unravel the epistemological and cognitive components of the concept of ‘competence’. 

This is, indeed, what pharmacists have done, as Antonio Sánchez Pozo has shown, writing in this collection of articles. They promote the competences deemed essential and desirable by professional consensus within their own scientific field. Their perception of competence derives from deontology, from the relevant knowledge base, and from the experience of professional practice. Their approach is principled and pragmatic. Rarely do they linger on definitions. Other criteria are more important: fulfilling prescribed ratios of theory to practice; satisfying the Competent Authorities that curricula are appropriate bases for registration; ensuring that the competences instilled in basic training can be clearly built upon and self-assessed in continuing professional development (CPD). 

## 8. What Are the Next Steps in Competence-Based Curricula?

There remain high levels of graduate unemployment in numerous European countries [22] (pp. 182–208). Given that the bulk of students accessing higher education come directly from the secondary sector, there is also a need for renewed commitment to competence-based education in secondary education. This much was revealed by the 2015 report of the Programme of International Student Assessment (PISA). This was the first time that all EU MSs had participated simultaneously. The headline conclusion drawn by the European Commission makes dismal reading:

When it comes to progress towards the 2020 benchmark of less than 15% low achievers, the EU as a whole is seriously lagging behind in all three domains and has taken a step backward, compared to the PISA 2012 results (science: 20.6%, +4.0 percentage points; reading: 19.7%, +1.9 percentage points; maths: 22.2%, +0.1 percentage point). Low achievers cannot successfully complete basic tasks that are required in modern societies and the consequences of this underachievement, if it is not tackled successfully, will be eminent and costly in the long run for them individually, but also for societies as a whole.

[23]

This unwelcome stimulus to policy-makers, with its negative implications for future higher education participation rates, concerns both those in the EU and those in the Bologna Process countries. Major policy shifts in the EU will be apparent by 2018, if they are to manifest themselves in programme opportunities in 2020. The next summit of the Bologna ministers is also scheduled for 2018, in Paris. What, at this stage, are their likely agendas?

In fact, the Commission has already adopted its *New Skills Agenda* [24]. It consists of ten actions to be implemented before the end of 2017. Most relevant to the present discussion are:

- a review of the Key Competences Recommendation, with a focus on skills acquired in non-formal and informal settings;
- a proposed Blueprint for sectoral cooperation on skills, which will identify skills gaps, assess their impact and develop strategies based on business-education partnerships; pilot work has already begun in six sectors, and the healthcare sector will follow later in 2017;
- a review of the EQF, designed to strengthen and broaden it, specifically by accelerating the process of referencing to it the national qualifications frameworks of EU and non-EU countries.

The review of the EQF has particular significance for pharmacists, as suggested in the final section of this article.

Much of the Commission’s thinking is underpinned by the ambitious European Skills, Competences, Qualifications and Occupations venture (ESCO), which aims to map onto the EQF the detailed taxonomy of occupations developed by the International Labour Organisation (ILO). The outcome will be a fully-functioning multilingual website designed to facilitate ‘competence-based job-matching’. Readers will be curious to discover the ESCO profile of pharmacy. It is a case of ‘watch this space’: the current online version is ESCO v0 [25], last updated in August 2014. 

The ESCO planners may consider that it is of particular usefulness to the regulated professions. The truth of the matter could be the other way round: it is the professions, with their tabulations of competences in context, and their translations of competences into curricula, which can inform ESCO and higher education policy-makers.

The Commission has recognised that the demographic and socio-economic back-drop has changed significantly since the days of the Lisbon Strategy. A high-skilled labour force remains necessary, but this is now due to the probable displacement of millions of low-skilled occupations by automation and digital technologies. High-skilled job creation has become the order of the day; loss of social cohesion has become the greatest perceived risk.

In the Bologna Process, meanwhile, thoughts have turned to ‘new policy goals’ and a working group has been set up to carry this preoccupation forward. It is not wholly clear what will eventuate in the field of competences. It may be that the focus will shift back to citizenship, in the light of the advances made by resurgent nationalisms and parties of the extreme right. If this is the case, the Council of Europe’s recent work on ***competences*** *for democratic culture* [26] will be a central feature. 

Such a shift would act as a pendant to the EU’s strong focus on labour market needs. The challenge for higher education systems and institutions would then be to find ways of implementing the two policy imperatives and of designing curricula which deliver both sets of competences in synergy. 

The next review of the Directive is scheduled for 18 January 2019. Article 60.2 specifies that the review will report on, *inter alia*, ‘the modernisation of the knowledge, skills and **competences** for the professions covered by Chapter III of Title III, [the seven sectoral professions] including the list of **competences** referred to in Article 31(7)’ [general care nurses]; and ‘the functioning of the common training frameworks and common training tests’. 

## 9. Conclusions

In conclusion, it is useful to return to the proposed review [27] of the EQF. If the review proceeds as the Commission intends, its outcomes will figure in the next review of the Directive; they will emphatically confirm that EU higher education policy has primarily a labour market focus; and they will also therefore inflect discussions within BFUG.

What, then, is intended? If the Recommendation is adopted, it will:

- cover all qualifications, including private-sector, non-formal and international qualifications;
- develop a standard format for the expression of a learning outcome;
- attach learning outcomes to ECTS in a more systematic manner;
- include as many third country qualifications frameworks as possible;
- strengthen the governance of the EQF.

In respect of competence, it will take steps to enhance the clarity of its terminology. In its tabulation of levels, the word ‘competence’ will be replaced by ‘responsibility and autonomy’. The next two years represent a window of opportunity for the pharmacists to finalise, implement and report on their competence-based curricula, in order to inflect positively any further amendments to the Directive and to impose their presence on higher education policy-makers.

## Appendix A. Incidence of the Word ‘Competence’ in Bologna Ministerial Communiqués.

Bologna 1999	A Europe of Knowledge is now widely recognised as an irreplaceable factor for social and human growth and as an indispensable component to consolidate and enrich the European citizenship, capable of giving its citizens the necessary **competences** to face the challenges of the new millennium, together with an awareness of shared values and belonging to a common social and cultural space.
Prague 2001	[no mention]
Berlin 2003	Ministers encourage the member States [i.e., the Bologna signatory countries] to elaborate a framework of comparable and compatible qualifications for their higher education systems, which should seek to describe qualifications in terms of workload, level, learning outcomes, **competences** and profile. They also undertake to elaborate an overarching framework of qualifications for the European Higher Education Area.
Bergen 2005	We adopt the overarching framework for qualifications in the EHEA, comprising three cycles (including, within national contexts, the possibility of intermediate qualifications), generic descriptors for each cycle based on learning outcomes and **competences**, and credit ranges in the first and second cycles. […] The European Higher Education Area is structured around three cycles, where each level has the function of preparing the student for the labour market, for further **competence** building and for active citizenship.
London 2007	Higher education should play a strong role in fostering social cohesion, reducing inequalities and raising the level of knowledge, skills and **competences** in society.
Leuven 2009	Student-centred learning and mobility will help students develop the **competences** they need in a changing labour market and will empower them to become active and responsible citizens. […] Lifelong learning involves obtaining qualifications, extending knowledge and understanding, gaining new skills and **competences** or enriching personal growth. […] With labour markets increasingly relying on higher skill levels and transversal **competences**, higher education should equip students with the advanced knowledge, skills and competences they need throughout their professional lives.
Budapest-Vienna 2010	We acknowledge the key role of the academic community—institutional leaders, teachers, researchers, administrative staff and students—in making the European Higher Education Area a reality, providing the learners with the opportunity to acquire knowledge, skills and **competences** furthering their careers and lives as democratic citizens as well as their personal development.
Bucharest 2012	Today’s graduates need to combine transversal, multidisciplinary and innovation skills and **competences** with up-to-date subject-specific knowledge so as to be able to contribute to the wider needs of society and the labour market. […] Lifelong learning is one of the important factors in meeting the needs of a changing labour market, and higher education institutions play a central role in transferring knowledge and strengthening regional development, including by the continuous development of **competences** and reinforcement of knowledge alliances.
Yerevan 2015	Thanks to the Bologna reforms, progress has been made in enabling students and graduates to move within the EHEA with recognition of their qualifications and periods of study; study programmes provide graduates with the knowledge, skills and **competences** either to continue their studies or to enter the European labour market; institutions are becoming increasingly active in an international context; and academics cooperate in joint teaching and research programmes. […] By 2020 we are determined to achieve an EHEA where our common goals are implemented in all member countries to ensure trust in each other’s higher education systems; where automatic recognition of qualifications has become a reality so that students and graduates can move easily throughout it; where higher education is contributing effectively to build inclusive societies, founded on democratic values and human rights; and where educational opportunities provide the **competences** and skills required for European citizenship, innovation and employment. […] Study programmes should enable students to develop the **competences** that can best satisfy personal aspirations and societal needs, through effective learning activities. […] We need to ensure that, at the end of each study cycle, graduates possess **competences** suitable for entry into the labour market which also enable them to develop the new **competences** they may need for their employability later in throughout their working lives. […] We will promote international mobility for study and placement as a powerful means to expand the range of **competences** and the work options for students. […]

## Appendix B. Definitions of ‘Competence’ in Successive Editions of the ECTS Guide.

ECTS Users’ Guide 2005	Learning outcomes are sets of **competences**, expressing what the student will know, understand or be able to do after completion of a process of learning, long or short. […] **Competences** represent a dynamic combination of attributes, abilities and attitudes. […] **Competences** are formed in various course units and assessed at different stages. They may be divided in subject-area related competences (specific to a field of study) and generic competences (common to any degree course).
ECTS Guide 2009	In Europe a variety of terms relating to “learning outcomes” and “**competences**” are used with different shades of meaning and in somewhat different frames of reference. In all cases however they are related to what the learner will know, understand and be able to do at the end of a learning experience. [The Guide cites the EQF definition of competence quoted earlier in this article, but goes on to provide the following further definition in its Glossary…] **Competence**: A dynamic combination of cognitive and metacognitive skills, knowledge and understanding, interpersonal, intellectual and practical skills, ethical values and attitudes. Fostering competences is the object of all educational programmes. Competences are developed in all course units and assessed at different stages of a programme. Some competences are subject-area related (specific to a field of study), others are generic (common to any degree course). It is normally the case that competence development proceeds in an integrated and cyclical manner throughout a programme.
ECTS Guide 2015	[The 2015 Guide retains the 2009 Glossary entry, itself based on the EQF definition of competence, but—in an attempt to disentangle competence from learning outcome—states that]… Learning outcomes express the level of **competence** attained by the student and verified by assessment.
